# The ‘National Finals Revision Day’ Teaching Strategy: A Cost-Effective Way to Pass Medical School ‘Finals’ and Upskill Junior Doctors

**DOI:** 10.7759/cureus.8977

**Published:** 2020-07-03

**Authors:** Alexander Curtis, Gregory Neal-Smith, Joseph T Fennelly, Richard Goodall, Edward Hayter, Charles Coughlan, Adam Miller, Daniel Huntley, Agnes Hamilton-Baillie, Henry M Drysdale, Katherine Hurrell, Will Hughes

**Affiliations:** 1 Trauma and Orthopaedics, Royal United Hospital, Bath, GBR; 2 Trauma and Orthopaedics, Oxford University Hospitals NHS Foundation Trust, Oxford, GBR; 3 Surgery and Cancer, Imperial College London, London, GBR; 4 Trauma and Orthopaedics, North Middlesex University Hospital, London, GBR; 5 Paediatrics, Imperial College Healthcare National Health Service Trust, London, GBR; 6 General Practice, Queen Elizabeth Hospital, Gateshead, GBR; 7 Trauma and Orthopaedics, Imperial College London, London, GBR; 8 Paediatric Orthopaedic Surgery, University Hospitals Bristol NHS Foundation Trust, Bristol, GBR; 9 Paediatric Surgery, University of Oxford, Oxford, GBR; 10 Emergency Medicine, Middlemore Hospital, Auckland, NZL; 11 Plastic Surgery, Mid and South Essex NHS Foundation Trust, Broomfield, GBR

**Keywords:** revision, course, finals

## Abstract

Courses to help medical students pass ‘Finals’ already exist but are typically expensive or can only be attended by a limited number of students. We describe the success of ‘The National Finals Revision Day' (NFRD) course, which we believe is sustainable and unique in terms of its combined scale and cost (£10 per person). The course was organised and taught by 12 junior doctors. In total, 300 students attended from 55% of UK medical schools. Attendees found the course both relevant (96.4%) and cost-effective (97%), whilst the 11 medical and surgical talks were of a high standard (90.1%). The organising committee felt confident to organise their own teaching course in the future with 100% having already run a course themselves since the NFRD course. The NFRD course was also used by 11/12 (91.7%) of the organising committee to achieve their Annual Review of Competency Progression (ARCP) and 12/12 (100%) of the organising committee to obtain jobs on training programmes in the UK. We provide guidance about how to organise similar large-scale events for those interested. Moving forward, the teaching course will be run at: (i) multiple times; (ii) multiple UK venues; (iii) run over two days to cover more medical and surgical topics; and (iv) include the option of attending via video link.

## Introduction

Medical school ‘Finals’ test a wealth of knowledge gained throughout medical school, acting as the final hurdle before starting life as a newly qualified doctor. This has been shown to be the most stressful period at medical school after investing so much time and money in the preceding years [1]. Courses already exist to help medical students pass ‘Finals’; however, many of the established courses are either expensive, charging over £130 ($171), or have limited participant capacity so cannot be attended by all [2].

The General Medical Council (GMC): Good Medical Practice recommends that qualified doctors should take on the role of mentor for more junior colleagues [3]. There is strong evidence that recently qualified junior doctors can provide high-quality teaching by offering a unique perspective for final year medical students, whilst also empowering the trainer with crucial teaching skills [4,5]. In addition, management and leadership skills of junior doctors can be developed as part of the organisation of a course, both of which form important areas of medical and surgical applications in the UK [6,7]. Our aim was to, therefore, provide a cost-effective, large-scale ‘Finals’ revision day that was available to all, whilst also providing opportunities to develop the teaching and management skills of the committee involved.

## Materials and methods

For the fourth consecutive year, a group of 12 junior doctors, ranging from Foundation Year One to Core Surgical Year Two (one to four years post-graduate experience, respectively) organised and gave a series of lectures to final year medical students, entitled the National Finals Revision Day (NFRD). The 2018 lecture series was held on 2nd December at the John Radcliffe Hospital, Oxford.

Planning started six months in advance and ticket sales commenced three months before the event. For the fourth year the cost remained £10 ($13) per person which included: (i) interactive talks on medical/surgical topics that are commonly examined in ‘Finals’; (ii) access to all teaching materials; (iii) weekly online questions (multiple choice questions); and (iv) tea/coffee breaks and lunch. The selected venue had a large capacity and was located in a central UK region with good transport links.

In total, 11 lectures were given by the organising committee: six medical and five surgical lectures. Each lasted 30 minutes, followed by a five-minute break, in addition to a longer morning break, lunch break and afternoon break. The specialties covered included: cardiology, respiratory, endocrine, neurology, gastrointestinal, renal, general surgery one, general surgery two, urology, vascular and emergencies. Each of the organising committee taught a topic relevant to their current or previous job and/or their career aspiration. The organising committee also completed tasks including advertising, catering, venue hire, sponsorship, ticket sales and helping on the day. The advertisement strategy utilised a broad range of methods including social media platforms, as well as contacting medical schools directly and distributing emails to their relevant mailing lists. One key feature of the advertisement strategy was to utilise the widely dispersed committee, with each member establishing a relationship with their regional medical school. This network allowed a large number of medical students to be contacted, and the regional representative was able to address any problems arising in the area.

Feedback was obtained both from the attendees and organising committee. This included a combination of Likert scale 1-5 responses and Yes/No responses [8]. Questions for the attendees focused on the quality and value of the event, whilst questions for the organising committee focused on teaching skills and management skills gained, and how these could be applied in the future.

## Results

In total, 300 final year medical students attended from 18/33 (55%) GMC approved UK medical schools. Ticket sales showed excellent growth over each of the four years: 101 (2015), 127 (2016), 145 (2017) and 300 (2018). Of those attending, 27.2% were from the local medical school, with the other 72.8% travelling from further away, which included 10% travelling >170miles each way to attend.

Online feedback was completed by 204 (68%) of students and highlighted: (i) the relevance to Medical and Surgical ‘Finals’ with attendees scoring this 4.82/5 (96.4%); and (ii) the excellent value for money with attendees scoring it 4.85/5 (97%). The mean score of the talks was 4.51/5 (90.1%) with a range of 3.79 to 4.78.

All of the organising committee completed feedback and 11/12 (91.7%) agreed that they had learned new teaching strategies as well as 12/12 (100%) feeling confident to teach in a large group setting. In addition, 12/12 (100%) felt that they had defined managerial roles allocated to them. Leading on from this, the organising committee felt very confident to organise their own event in future scoring this 4.4/5 (range 3-5), with all of the organising committee (100%) having already run a course themselves since the NFRD course. The NFRD course was also used by 11/12 (91.7%) of the organising committee to achieve their Annual Review of Competency Progression (ARCP) and 12/12 (100%) of the organising committee to obtain jobs on training programmes in the UK.

## Discussion

For the fourth consecutive year, we have provided a cost-effective revision course that can be used to supplement other forms of revision to help support students as they approach ‘Finals’. We are not aware of any other course that caters to so many students at such a competitive price whilst maintaining very high feedback. Year-on-year ticket sales have increased, confirming that the course is both sustainable and likely being recommended amongst students. It is important to note that this course is only one revision tool, with each lecture lasting only 30 minutes. It was therefore not possible to cover all topics that can be tested in 'Finals'. For this reason, the NFRD course should not be used in isolation, but to inform students of strengths as well as gaps in their knowledge.

For those interested in running similar events that keep the cost for those attending minimal we offer the following advice: (i) start planning the event at least three to six months in advance, making sure that there are no other competing courses due to run at the same time as this will dilute your audience participation; (ii) sponsorship is crucial to reduce costs. Contacting potential sponsors (both medical and non-medical) early increases the chance of success as most sponsors have an annual sponsorship cap [9,10]. Offering different sponsorship packages can be attractive to sponsors, for example, we selected ‘Bronze’, ‘Silver’ and ‘Gold’ packages which include company logo on handouts/merchandise, stall at the event and giving a short talk at the event, respectively. This followed advice from sponsorship companies [11]. Alternatively, ‘à la carte’ sponsorship is an increasingly popular sponsorship strategy that focuses on the individual needs of the sponsors rather than providing them with restrictive packages [12]. If you are struggling to identify potential sponsors, there are also companies that allow you to pitch your event globally, and interested sponsors from both the medical community and non-medical community can then approach you [13]; (iii) Catering costs vary hugely from as little as £2 to £10 per person ($2.60 to $13), so we would advise discussing costs with a range of caterers and liaise with colleagues who may work for companies that offer food discounts. Make sure that the caterer you select replies promptly to your messages, is flexible to your needs and has excellent customer reviews [14]. Certain food types can be provided by the organisers such as drinks, biscuits, crisps and fruit, with tea urns an excellent investment if planning multiple events in future; (iv) Venue costs can be very expensive so talk to your local medical school and see if they can offer a reduced rate as their medical students will benefit from the event; (v) It is increasingly important to utilise varied methods of advertisement to reach out and engage with students. As with other event organisers, we started advertising three months in advance and approached individual medical school surgical societies, medical societies, medical school administrators, and medical school Facebook groups [9]. The success of this advertising campaign is not only clear from the ticket sales we achieved, but also when you consider that 72.8% of attendees were not from the local medical school. In the future, we also plan to approach journals to advertise our events as they have a much larger student readership; and (vi) Encourage diversity amongst the organising committee. Our organising committee graduated from six different medical schools and we feel that this provided a varied insight into different techniques used to examine Medical School ‘Finals’ so that we could better cater to all students needs [15].

With competition for medical and surgical training numbers remaining high, it is imperative to score well on the self-assessment portfolio for each training programme [16]. The NFRD course provides maximum points in both the teaching and leadership/management sections of core surgical and core medical interviews [6,7]. The teaching and management skills obtained by the organising committee also acts as an alternative to attending widely available but expensive teaching and management courses that can cost in excess of £500 ($617) [17]. These teaching skills included learning new teaching styles for large group teaching, utilising voting apps to answer questions and reviewing feedback obtained from the attendees. The management skills included establishing an effective advertising campaign, cost-evaluation, sponsorship and on the day running of the course. This is particularly important with doctors increasingly taking on additional roles as teachers and managers in the clinical environment [18].

## Conclusions

The NFRD course is a cost-effective, large-scale revision course for final year medical students, receiving excellent feedback from attendees. It also offers important teaching and managerial skills to those involved with organising the event, both of which are crucial to success in specialty applications and more generally in the clinical environment. This course has gone through many iterations in the last four years, and we hope that by providing guidance on how to run a large-scale course, we will allow other enthusiastic junior doctors to establish similar courses that benefit students. For future courses, there are four important developments we are considering: (i) Running the course over two days (surgery one day, medicine one day) with the benefit of covering more material including rheumatology, infectious diseases, ENT and orthopaedics. This would be at the expense of students having to fund overnight accommodation and an increase in the course cost, which we estimate would rise to £15 ($18.50) to account for food and drink requirements; (ii) Run the course twice a year to benefit those who have finals at different times of year; (iii) Run a second course in the North of England to allow students in Northern medical schools to more easily access the course; and (iv) Include the option to attend the course via video link, which, given the current COVID-19 pandemic, may be of increasing relevance in the future.
